# Cincinnati Academy of Medicine

**Published:** 1877-02

**Authors:** 


### CINCINNATI ACADEMY OF MEDICINE.

### ANAL FISSURE.

Dr. Whittaker stated that he was consulted, a few days since, by a young lady, aged twenty-six, who complained of a continued pain about the rectum, which became quite excruciating during the act of defecation, and seemed to extend upward toward the diaphragm. The speaker was confident that the pain depended upon some local cause, as all remedies administered by other physicians had failed in relief. The slightest touch during examination gave her intense pain. It was impossible to introduce a speculum or the finger. On everting the anus he discovered, at the posterior border, a very small fissure with ulcerated edges. A free incision at its base gave the patient prompt and permanent relief. He mentioned the case, not because of its rarity, but for the purpose of getting an expression of views from the surgeons as to the best method of treating this peculiar affection, and because the pain produced by it was out of all proportion to the extent of surface involved.

Dr. Conner said that division of the base of the fissure is expected to be followed by a cure; the same result is attained, or at least temporary relief is given, if the base of the ulcer can be thoroughly separated from the underlying muscles. In addition to both these methods, the speaker had operated by rupture of the muscle with the fingers, by the application of nitrate acid, and of nitrate of silver; but expressed a preference for the use of the knife as the most radical and the least painful measure.

### BINOCULAR GLIOSARCOMA

Dr. Zinke's paper on this subject coming up for discussion, Dr. Longworth remarked that one of the most interesting clinical features of the case was the great length of time which intervened between the first manifestations of the tumor and the death of the child.

Until a recent date all tumors of this class were considered a variety of cancer; but this is not a cancer; it is a variety of sarcoma. Microscopical examination shows that it is made up almost entirely of small round cells like the cells found so abundantly in rapidly-growing embryonic tissue, or closely resembling the lymphoid cells and white blood corpuscles. These cells are so closely packed that they very nearly overshadow the intercellular substance, which, when brought to light by the removal of the cells from a microscopic section, with a camel's hair brush, appears in places hyaline and structureless, and in cases to consist of the finest and most delicate interlacement of fibres, resembling the fragile connective tissue which binds together the nerve elements of the brain and of the retina, the so-called neuroglia.

There may be such a thing as simple glioma of the retina, but usually round cells are found, as here, and it is to these that the tumor owes its malignancy. It is believed that the young sarcoma cells are endowed with properties similar to the lymph cells, and wandering through the lymph spaces and canals may establish colonies at a distance from the original tumor. Thus, though not a cancer, it is second to no form of cancer in malignancy.

As it is at first intra-ocular, experience showing that it begins in the retina, an early extirpation may prevent these metastatic deposits, so much to be dreaded. In its growth it may involve the different coats of the eye, but it usually escapes posteriorly along the line of the optic nerve.

Dr. Seely said that the fact of this being a non-inflammatory affection had led to a careful study of the exact location in which it originates, but as yet without any very positive results. Virchow believed that it originated in the outer granular layer of the retina; before this, Schweiger had concluded that the inner granular layer was the seat of its origin, and in this view he is supported by Hirschberg and Knapp. Iwanoff maintains that there are two varieties, one originating in the inner and the other in the outer granular layer. Visual disturbances very soon take place in the clinical progress of the cases, but as they usually occur in very early childhood, these are seldom of much diagnostic value, the blindness often not being discovered until late in the affection.

The time of its external appearance is variable. Hirschberg lays great stress on the point that rupture most frequently occurs early and posteriorly; whereas the fact is that when rupture does take place it is most likely to be anteriorly through the cornea; the next most common position is at the sclero-corneal junction.

The disease very often, as in this case, appears in both eyes, but seldom simultaneously. Clinical facts do not bear out the supposition of a transfer from the opposite eye through the optic chiasm. The well-known wandering properties of the cells of which this class of tumors is composed render it extremely important to remove the optic nerve as far back as possible; and the appearance or non-appearance of these cells in the cut extremity of that nerve may serve a valuable purpose in the prognosis as to the opposite eye.

Gliosarcoma is found in young children, often in several of the same family. If we have reason to believe that it has not extended along the optic nerve, a very early removal should be attempted.—*Clinic.*

